# Long-term aerobic and combined exercises enhance the satiety response and modulate the energy intake in patients with type 2 diabetes mellitus (T2DM): A randomized controlled trial

**DOI:** 10.1186/s13102-023-00655-x

**Published:** 2023-03-30

**Authors:** Dinithi Vidanage, Sudharshani Wasalathanthri, Priyadarshika Hettiarachchi

**Affiliations:** 1 Department of Nursing and Midwifery, Faculty of Allied Health Sciences, General Sir John Kotelawala Defence University, Rathmalana, 10390 Sri Lanka; 2 Department of Physiology, Faculty of Medicine, University of Colombo, Kynsey Road, Colombo 08, 00800 Sri Lanka; 3 Department of Physiology, Faculty of Medical Sciences, University of Sri Jayewardenepura, Gangodawila, Nugegoda, 10250 Sri Lanka

**Keywords:** Exercise, Appetite, Energy intake, Type 2 diabetes mellitus

## Abstract

**Background:**

Energy homeostasis plays a vital role in achieving glycemic control in people with type 2 diabetes mellitus (T2DM). Exercise is known to increase energy expenditure. However, its role in energy intake has not been explored in people with T2DM. Thus, this study aimed at determining the impact of long-term aerobic and combined exercises in modulating hunger, satiety and energy intake in T2DM.

**Methods:**

A randomized controlled trial, with 108 people with T2DM, aged 35–60 years were assigned into an aerobic, combined (aerobic and resistance) and a control group. Primary outcomes were subjective levels of hunger and satiety measured by a 100 mm visual analogue scale in relation to a standard breakfast meal (453 kcal) and energy and macronutrient intake determined by a 3-day diet diary at 0, 3 and 6 months.

**Results:**

Aerobic and combined groups exhibited reduced hunger and increased satiety at 3 and 6 months (*p* < 0.05). The combined group demonstrated a profound increase in satiety at 3 and 6 months compared to aerobics (3 months; *p* = 0.008, 6 months; *p* = 0.002) and controls (3 months; *p* = 0.006, 6 months, *p* = 0.014). Mean daily energy intake was reduced only at 6 months in the aerobic group (*p* = 0.012), whereas it was reduced in the combined group at 3 and at 6 months compared to controls (3 months: *p* = 0.026, 6 months: *p* = 0.022).

**Conclusions:**

Long-term aerobic and combined exercises produced a reduction in hunger, energy intake and increase satiety in people with T2DM. Despite energy expenditure, exercise seems to play a significant role in reducing energy intake as well. Combined exercises show more advantages over aerobic exercise since combined exercises have a greater impact on satiety and energy intake in people with T2DM.

*Trial registration number*: SLCTR/2015/029, https://slctr.lk/trials/slctr-2015-029.

## Background

The prevalence of type 2 diabetes mellitus (T2DM) and its co-morbidities have heightened to epidemic proportions across the globe in developing [1, 2] as well as in developed countries [3]. Physical inactivity and unhealthy food habits are important modifiable risk factors determining the progression of the disease. Adjusting to a new lifestyle by engaging in physical exercises and consuming a healthy diet plays a significant role in achieving optimal glycemic control [4]. Physical exercises comprise aerobics such as running, swimming, brisk walking, etc., or resistant types such as pushups, lifting weights, stretching exercises or yoga, etc., or a combination.

Combined exercises (aerobic and resistance together) are reported to be more beneficial for people with T2DM since those exercises produce a better glycemic control compared to aerobic or resistance exercises alone [5, 6]. Recent reports suggest that combined exercises for more than 150 min per week reduce HbA1c levels by about 0.9% in people with T2DM [7].

Emerging evidence indicates that besides energy expenditure physical activity plays a significant role in energy intake as well [8, 9]. Recently it was reported that an acute bout of exercise can influence the subsequent energy intake in healthy individuals [10]. The individuals may consume more than they would normally eat as they anticipate future energy expenditure or overestimate the amount of energy they have expended from physical activity. This licensing effect of food consumption is likely to affect energy intake in active individuals [11].

However, a previous study by Muller et al. [12], failed to show any change in energy intake following a single bout of aerobic exercise in subjects with T2DM despite having elevated levels of satiety for up to 3 h.

These conflicting results of energy intake in acute bouts of exercise indicate that short-term exercises do not have much value in appetite regulation. Indeed, as shown by a recent review, energy intake and hormonal fluctuations are short-lived with a single bout of exercise in healthy individuals [13]. However, the impact of habitual or long-term physical activities on health may differ from short-term exercise. Healthy adults engaging in long-term exercises had a greater satiety response despite eating more food to meet the energy deficits [8, 14].

Most interventional studies assessing appetite markers and gut hormones in long-term exercises were carried out in normal weight and overweight /obese healthy adults [15, 16]. Yet the impact of long-term exercises on hunger, satiety and energy intake in people with T2DM has not been explored systematically to date. Thus, we hypothesized that long-term exercises modulate the appetite markers and energy intake in people with T2DM. Therefore, this clinical trial aimed at determining the impact of home-based long-term aerobic and combined exercises on appetite markers and energy intake in people with T2DM.

## Methods

This is a randomized controlled trial (parallel-group trial design with an allocation ratio of 1:1) conducted at the research laboratory, Department of Physiology, Faculty of Medical Sciences, University of Sri Jayewardenepura, Sri Lanka. This trial was first registered at the Sri Lanka Clinical Trial Registry on 16/12/2015, (Registration number—SLCTR/2015/029). The trial consisted of two phases i.e., phase I (assessment of taste perception) and phase II (assessment of appetite markers and energy intake).

### Sample size

Nearly 300 participants were screened for medical illnesses, diet and regular exercises of which, only 225, participants who fulfilled the inclusion criteria were recruited for the study. A total of 225 participants were assigned to an aerobic exercise group, a combined exercise group and age, sex, body mass index (BMI)-matched control group by simple randomization. The appetite markers were assessed in a matched sub-sample of 108 participants (Aerobic group = 36, Combined group = 36, Control group = 36). The sub-sample size was calculated by using a randomized controlled trial sample size formula (2-tailed test) with type one (α) and type two (β) errors of 0.05 and a power of 80% [17]. The dropout (15%) observed in phase I of this trial was used to calculate the sub-sample. Mean values of HbA1c % (in phase I) were used as the key variable for sample size calculation [18].

### Randomization

The allocation (1:1 ratio) was done using a random number table. An arbitrary number was chosen from the random number table, which is a 3-digit number (since the total sample size carries a 3-digit number). The randomly picked number was matched with the participant’s serial number. Thus, the 1st matched number was assigned to the aerobic group, the 2nd matched number to the combined group and the 3rd matched number to the control group. Enrolling participants and assigning them to the 3 groups were done by the principal investigator.

### Participants and eligibility criteria

Participants aged 35–60 years with a history of T2DM attending the Family Practice Centre of the University of Sri Jayewardenepura, Sri Lanka were recruited for this study from October 2016 to January 2018. People with T2DM were recruited every Tuesday and Thursday at the clinics held at the Family Practice Centre, University of Sri Jayewardenepura. To invite participants to the study, a notice comprising eligibility criteria, benefits of the study and the contact details of the principal investigator was displayed on the notice board at the clinic premises. To increase visibility for those who were waiting for clinic registration, the notice was placed beside the wall adjacent to the patient registration counter.

The 3 arms of the trial included 2 intervention groups i.e., aerobic exercise, a combined exercise group (aerobic and resistance exercise) and age, sex, BMI matched control group. Written informed consent was obtained from the eligible participants before recruitment for the study. People with T2DM who were inactive at the time of recruitment were enrolled for all three groups of the study. i.e., the two exercise groups, aerobic and combined and the controls. Based on previous literature [19], people who have not engaged in at least one exercise session (1 session = 40 min or more of moderate to vigorous intensity exercises) during the past 6 months were considered inactive. People with T2DM aged between 35 and 60 years, diagnosed with a history of T2DM for more than 5 years, with glycated haemoglobin (HbA1c) between 6.6 and 9.9% and those who were willing to perform regular exercises and those who were willing to maintain a 3-day diet diary were included in the study.

Participants with ischemic heart disease, uncontrolled hypertension (systolic blood pressure > 160 mmHg), psychiatric illnesses (mood changes, schizophrenia, etc.) [20] and, physical and neurological disorders that affect exercises were excluded from the study. People with T2DM who were on medications known to influence taste (insulin, sulfonylureas, etc.) [21, 22], those with diseases of the oral cavity, smokers, and those on special therapeutic diets were excluded from the study.

**Fig. 1:**
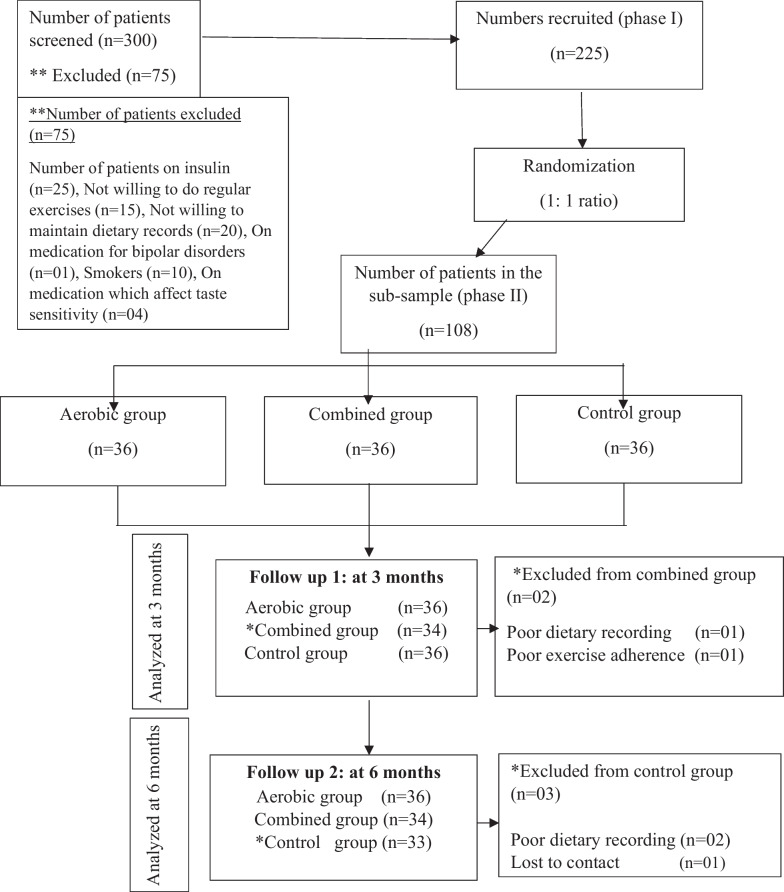
Flow diagram showing the outcome of recruited participants as per CONSORT (2010) guidelines

The accuracy of study instruments was ensured by a pilot study conducted with 10 participants who fulfilled similar inclusion and exclusion criteria. A resting ECG was performed and a detailed medical history was obtained from the participants of the intervention groups to assess their cardiovascular fitness for exercises. Figure 1 shows a flow chart indicating the outcome of recruited participants.

### Outcomes

Primary outcomes were the subjective level of hunger and satiety, mean daily energy and macronutrient intake (carbohydrate, protein and fat) assessed at 3 and 6 months.

The secondary outcome was the HbA1c level assessed at 6 months.

### Data collection

Data were collected in batches of 5–6 participants per day who were instructed to attend on a predetermined date to avoid contamination. Participants were instructed to abstain from food and beverages from 10 pm the previous day and to have 6–8 h of sleep and on the following morning, only tea/coffee (without sugar and milk) was allowed. On the day of the tests, the participants arrived at the research laboratory between 08.00 and 8.30 am. Baseline sociodemographic, clinical and anthropometric characteristics were recorded on arrival. They were requested to return the 3-day diet diary at each follow-up visit. Five milliliters (5 ml) of blood were drawn under strict aseptic conditions for the determination of HbA1c by the high-performance liquid chromatography method. Subjective levels of hunger, satiety, energy and macronutrient intake, and anthropometric measurements (body weight, height) were assessed at baseline, at 3 and 6 months in all groups. Participants were requested to report to the principal investigator, about any significant change in medication/diet, during the study period.

All stages of data collection were done by the principal investigator and the treating physician at the clinic did not have any involvement in this process.

### Assessment of anthropometry

Anthropometry was assessed at baseline, at 3 and at 6 months. Height was measured using a mechanical stadiometer (Seca-213). To measure height, the  participants were asked to remove footwear and stand up straight against the backboard ensuring contact of the backside of the head, shoulder blades, buttocks and heel with the backboard. Height was measured to the nearest 0.1 cm. Weight was measured by a digital flat weighing scale (Seca-803). Weight measurements were taken in patients without shoes. They were instructed to step on and stand on the center of the scale with hands hanging lateral to the body while looking straight. Weight measurement was taken to the nearest 0.1 kg.

BMI was calculated using weight and height. BMI of less than 18.5 kg/m^2^ is classified as underweight, 18.5–22.9 kg/m^2^ as normal, 23–24.9 kg/m^2^ as overweight and equal to or more than 25 kg/m^2^ as obese according to Asian standards [23].

### Assessment of subjective level of hunger and satiety

Hunger and satiety were assessed using a 100 mm visual analogue scale (VAS) which is reliable for appetite research [24]. The participants were instructed to rate the subjective levels of perceived intensity of hunger and satiety on the VAS 30 min prior and, 30 and 60 min after a standard breakfast meal i.e., 3 slices of brown bread (30 g each/76 kcal) added with a tablespoon of margarine (20 g/140 kcal) and a banana (100 g/95 kcal). The amounts of each nutrient were not normalized for each subject’s body weight as the body weight of participants at baseline was not significantly different between groups. All participants in the intervention and control groups were given the same standardized meal comprising of the same nutrients prior to the assessment of appetite parameters.

The subjective level of hunger and satiety were assessed based on simple questions, “How hungry do you feel right now?”, “How pleasant would it be to eat right now?”, “How much do you think you could eat right now?” and “How full do you feel right now?”. As sickness (i.e., nausea/vomiting, fever, sore throat, etc.) affects the appetite, the subjective rating of ‘feeling sick’ was also assessed using the VAS.

### Assessment of energy and macronutrient intake

A validated Sinhala version of a three-day diet diary was used to determine the dietary behaviors of the participants [25]. This diary comprised guidelines for describing dietary practices e.g., type of food, method of cooking, the method for recording the quantity of food (i.e., pictures of portion sizes measured using a teaspoon, tablespoon, cup), etc.

Energy and macronutrient intake were determined at 0, 3 and 6 months in the intervention and the control groups by a 3-day diet diary, as it has been reported a better tool compared to 24-h dietary record and 5-day food frequency questionnaire [26]. The participants were not specifically instructed on their diet. They were instructed to record their usual diet on two weekdays and one day on the weekend and were instructed to avoid days that the usual meal was changed (e.g. illnesses, outings, etc.).

A sample dietary record including the time of the meal, type of food, method of cooking and the portion size with relevant instructions was provided to minimize errors in recording. The diet diaries were collected by the principal investigator at each follow-up visit. The energy and macronutrient intake were calculated using Nutrisurvey 2007 software-EBIS Pro, Germany [27, 28]. The total amount of energy calculated in the 3-day diet diaries was averaged for each participant to obtain the mean daily energy intake.

The participants who did not provide the 3-day diet diaries at follow-ups and those who provided incomplete diet diaries were considered “poor dietary recording”.

### Exercise intervention

A graded exercise protocol [29] was introduced to the participants in the intervention groups. A prior exercise session was conducted at the Department of Physiology to familiarize the the exercise protocols with the participants. The practice sessions were conducted on two separate days for aerobic and combined groups to avoid contamination. The aerobic group was instructed to perform brisk walking (moderate intensity) for a minimum of 150 min/week spanning over 3–5 days for 6 months. The combined exercise group was instructed to perform stretching exercises for 5 major muscle groups (i.e., biceps, triceps, hamstring, quadriceps, pectoralis major) using resistance bands (1.5 m length), 20 min/day, 2–3 days/week for 6 months in addition to the aerobic exercise protocol. The participants were instructed to select either a home-based setting or an outdoor walking track to perform aerobic exercises and resistance exercises and also informed about the possible adverse effects expected during/after exercises [i.e., hypoglycemia, exercise-related acute physical injuries, symptoms of myocardial ischaemia (chest pain/angina, burning discomfort, exertional dyspnoea, increasing fatigue, sweating, light-headedness, nausea)] [30] where they should not continue the exercise session.

No financial intensives were given to participants to comply with the exercises. However, they were given the blood investigation (HbA1c) done free of charge, which is an expensive investigation in Sri Lanka. Further, the pedometers, resistance exercise bands and breakfast for each participant were given free of charge. Special instructions regarding exercises were not provided for the control group.

### Assessment of adherence to exercises

To ensure accurate evaluation of adherence to exercises, each participant in the intervention group was provided with a ‘pedometer’ that indicates the step count, the distance walked and the number of calories burnt. Participants were instructed to wear the pedometer during aerobic exercise sessions and note down the readings on a given paper after each exercise session.

In addition, the participants in both intervention groups maintained exercise adherence charts recording the date, time, duration of exercise (minutes), number of days of exercises/week, etc. Participants who performed < 150 min of aerobic exercises/week or < 40 min of resistance exercises/week were excluded from the study. Adherence to exercise protocols was further ensured by telephone reminders in the 2nd week of each month. The control group was also contacted in the same way to find out whether they have commenced formal exercises during the study period. As none of the controls engaged in formal exercises, there was no need to exclude any of them from the study.

### Blinding

The blinding of the participants was not possible due to the nature of the intervention. Data collection of all stages in the study was done by the principal investigator of the study.

### Statistical analysis

Descriptive data of participants were reported as numbers and percentages or means/standard deviations (± SDs). Mean/median differences (Δ) in the hunger and satiety, energy and macronutrient intake were obtained by deducting the baseline values from 3- and 6-month values. Mean differences ± SD in the intervention groups for hunger and satiety were compared with that of the control group by one-way ANOVA followed by Tukey post-hoc test. Similarly, differences (median/IQR) between 3 and 6 months with the baseline values for energy and macronutrient intake were compared between the groups by Kruskal Wallis-H test followed by Freidman post-hoc.

Absolute values (mean ± SDs) of body weight and BMI at 3 months and 6 months between groups were compared by one-way ANOVA followed by Tukey post-hoc test. Similarly, absolute values of HbA1c at 6 months between groups were compared by one-way ANOVA followed by Tukey post-hoc test.

To assess the impact of regular exercises on hunger-satiety, energy and macronutrient intake, HbA1c, body weight and BMI over time, the absolute values of all dependent variables were compared by Two-way repeated-measures ANOVA [i.e., time (within-subject factor) and intervention (between-subject factor)] followed by Bonferroni post-hoc test. All data were analyzed using SPSS software version 23.0 considering the level of significance *p* < 0.05.

There were no missing data at the baseline. However, participants with missing data at 3 and at 6 months in the dataset were not considered for analysis.

### Ethical considerations

This study protocol was registered at the Sri Lanka Clinical Trial Registry (SLCTR/2015/029). The protocol was approved by the Ethics Review Committee (ERC approval no. 10/18) of the Faculty of Medical Sciences, University of Sri Jayewardenepura, Sri Lanka. Informed written consent was obtained from each participant at recruitment. Participants were permitted to withdraw from the study at any time. Each participant was provided with a serial number for identification and data entering purposes. All data were kept confidential in a password-protected computer. Only the research team had access to the data.

## Results

Baseline sociodemographic, clinical and anthropometric characteristics of the study participants are shown in Table 1. There was no significant difference in the mean age, gender and BMI between any of the groups. None of the participants reported any adverse effects expected during/after exercises [i.e., hypoglycemia, exercise-related acute physical injuries, symptoms of myocardial ischaemia (chest pain/angina, burning discomfort, exertional dyspnoea, increasing fatigue, sweating, light-headedness, nausea)]. Those who showed poor adherence to exercises were removed from the analysis as they have not adhered to the recommended exercise intervention. Moreover, the incompleteness of the diet diaries did not reflect the actual food intake of the study participants. Therefore, the principal investigator did not consider such data for analysis.

**Table 1:** Baseline characteristics of study participants

Variable	Aerobic group (n = 36)	Combined group (n = 36)	Control group (n = 36)	*p* value
Age (years) (mean ± SD)*	48.9 ± 6.4	52.8 ± 4.9	50.5 ± 5.0	0.12
Gender, n (%)				
Male	18 (50%)	23 (63.9%)	16 (44.4%)	0.138
Female	18 (50%)	13 (36.1%)	20 (56.6%)	
Duration of T2DM (years) (mean ± SD)*	8.8** ± **5.5	6.9 ± 2.4	8.3** ± **4.7	0.172
Family history of T2DM, n (%)				
Yes	22 (61.1%)	21 (58.3%)	25 (69.4%)	0.877
No	14 (38.9%)	15 (41.7%)	11 (30.6%)	
Diet control, n (%)				
Yes	21 (58.3%)	17 (47.3%)	22 (61.1%)	0.673
No	15 (41.7%)	19 (52.7%)	14 (38.9%)	
HbA1c (%)* (mean ± SD)	8.2** ± **1.9	7.2** ± **1.0	8.2 ± 1.6	0.18
Body weight (kg)* (mean ± SD)	60.0** ± **9.3	60.7** ± **7.6	60.3** ± **9.3	0.952
BMI (kg/m^2^)* (mean ± SD)	23.6 ± 4.0	24.9** ± **3.6	25.4** ± **3.0	0.102

^*^Mean ± SD compared by one-way ANOVA

Table 2 shows the pedometer readings obtained from participants in the intervention groups.

**Table 2:** Data obtained from pedometer in the intervention groups

	Data on pedometer		
	Step count during aerobic exercise	Energy expenditure during aerobic exercise (kcal)	Distance of walk (km)
*0–3 months (mean ± SD)*			
Aerobic	3614.0 ± 140.1	1775.7 ± 43.4	3.6 ± 0.3
Combined	3720.4 ± 653.3	1778.5 ± 41.2	3.8 ± 0.3
*3–6 months (mean ± SD)*			
Aerobic	3944.5 ± 371.6	1848.5 ± 46.3	3.7 ± 0.3
Combined	3998.8 ± 426.2	1853.5 ± 47.1	3.9 ± 0.3

^*^No data on the control group was shown in the table as controls were not engaged in formal exercises

**Fig. 2:**
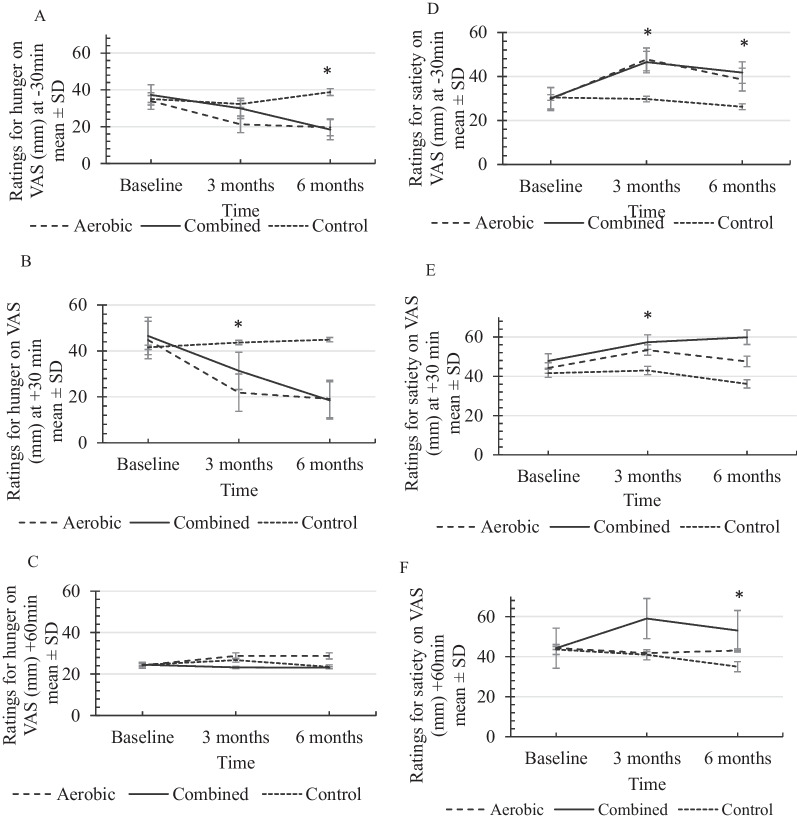
Perceived level of hunger (absolute ratings) shown as **A** (− 30 min), **B** (+ 30 min), **C** (+ 60 min) and perceived level of satiety (absolute ratings) shown as **D** (− 30 min), **E** (+ 30 min), **F** (+ 60 min) on 100 mm VAS between study participants assessed at 0,3 and 6 months in relation to a standard meal, compared by one-way ANOVA. Values are reported as mean ± SD, *p < 0.05

Commencing from the baseline up to 6 months, the hunger levels showed a decreasing trend in both intervention groups with a greater reduction in the combined exercise group at − 30 min and + 30 min. The level of satiety showed a more intense increase in the combined exercise group at all time points (− 30, + 30, + 60 min) (Fig. 2).

As indicated in Table 3, hunger levels were reduced at 3 months and 6 months at − 30 min and + 30 min time points in relation to a standard meal in the aerobic exercise group whereas in the combined exercise group hunger levels were reduced at − 30 min at 3 months and − 30 and + 30 min time points at 6 months in relation to a standard meal. These changes in the hunger levels at 3 and 6 months in relation to the baseline were significant compared to that of the control group.

**Table 3:** Comparison of mean differences of hunger and satiety between baseline, 3 and 6 months at selected time points in relation to a standard meal

Time	^#^Mean differences (Δ) ± SD between 0 and 3 months			*p* value			^¥^Mean differences (Δ) ± SD between 0 and 6 months			*p* value		
	Aerobic (AE) (n = 36)	Combined (COM) (n = 34)	Control (CON) (n = 36)	AE vs CON	COM vs CON	AE vs COM	Aerobic (AE) (n = 36)	Combined (COM) (n = 34)	Control (CON) (n = 33)	AE vs CON	COM vs CON	AE vs COM
*Difference in hunger levels*												
− 30 min	− 22.94 ± 3.80	− 15.85 ± 3.19	+ 2.10 ± 2.16	0.001*	0.018*	0.533	− 25.63 ± 5.31	− 28.73 ± 4.05	+ 3.63 ± 1.39	0.001*	0.001*	0.902
+ 30 min	− 12.63 ± 3.32	− 7.26 ± 9.35	− 2.71 ± 2.20	0.050*	0.556	0.455	− 14.29 ± 9.32	− 18.79 ± 8.36	+ 4.19 ± 1.07	0.001*	0.001*	0.527
+ 60 min	+ 4.51 ± 2.30	− 1.71 ± 2.34	+ 2.30 ± 1.17	0.939	0.761	0.929	+ 4.51 ± 2.13	− 1.83 ± 1.04	− 0.44 ± 1.85	0.061	0.944	0.133
*Difference in satiety levels*												
− 30 min	+ 18.00 ± 6.94	+ 15.91 ± 5.93	− 0.72 ± 1.74	0.025*	0.050*	0.956	+ 8.83 ± 3.09	+ 11.15 ± 3.77	− 4.51 ± 2.86	0.012*	0.049*	0.934
+ 30 min	+ 9.11 ± 2.75	+ 10.81 ± 3.53	+ 1.41 ± 2.04	0.383	0.023*	0.957	+ 3.36 ± 1.63	+ 12.14 ± 2.21	− 5.65 ± 2.14	0.409	0.050*	0.522
+ 60 min	− 2.53 ± 1.67	+ 17.71 ± 3.57	− 2.64 ± 1.42	0.998	0.006*	0.008*	− 12.24 ± 2.33	+ 7.52 ± 1.56	− 8.5 ± 6.22	0.776	0.014*	0.002*

Compared by Tukey post-hoc test, *p < 0.05

^#^Values were obtained by deducting the baseline value from the 3 months

^¥^Values were obtained by deducting the baseline value from the 6 months

As shown in Table 3, satiety levels were increased at 3 months and 6 months at − 30 min in relation to a standard meal in the aerobic group whereas the satiety levels in the combined group were increased at all time points in relation to a standard meal at 3 months and 6 months. These changes in the satiety levels at 3 and 6 months in relation to the baseline were significant compared to that of the control group.

Two-way repeated-measures ANOVA showed a significant time × group interaction (Wilk’s Lambda) at − 30 min [F = (4,206) = 7.0, *p* = 0.001] and + 30 min [F = (4,204) = 8.9, *p* = 0.001] for the perceived level of hunger.

Bonferroni post hoc comparisons confirmed that the interactions of time × intervention for hunger levels were significant in both exercise groups at − 30 min [aerobic vs control: − 14.5 mm, CI = − 20.4, − 8.6, *p *= 0.001; and combined vs control: − 10.7 mm, CI = − 4.8, − 16.7, p=0.001], and + 30 min [aerobic vs control: − 10.4 mm, CI = − 5.2, − 15.5, *p* = 0.001 and; combined vs control: − 6.8 mm, CI = − 1.5, − 12.0, *p* = 0.001] in relation to a standard meal where both exercise groups showed significantly reduced hunger levels compared to controls.

Similarly, two-way repeated measures ANOVA showed significant time × group interaction (Wilk’s Lambda) at − 30 min [F (4,204) = 2.6, *p* = 0.036], + 30 min [F (2,102) = 3.3, *p* = 0.041] and + 60 min [F (4,204) = 3.0, *p* = 0.018] for the satiety levels.

Bonferroni post-hoc comparisons revealed that the interaction of time × intervention for perceived satiety was significant between aerobic vs control: 9.8 mm, CI = 16.9, 2.6, *p* = 0.004 and combined vs control: 10.3 mm, CI = 18, 3.4, *p* = 0.002, 30 min before a standard meal. In addition, the interactions (time × intervention effect) were significant between aerobic vs control: 7.9 mm, CI = − 15.4, 0.5, *p* = 0.032 and combined vs control: 14.9 mm, CI = 22.4, 7.3, *p* = 0.001 30 min after the meal.

**Fig. 3:**
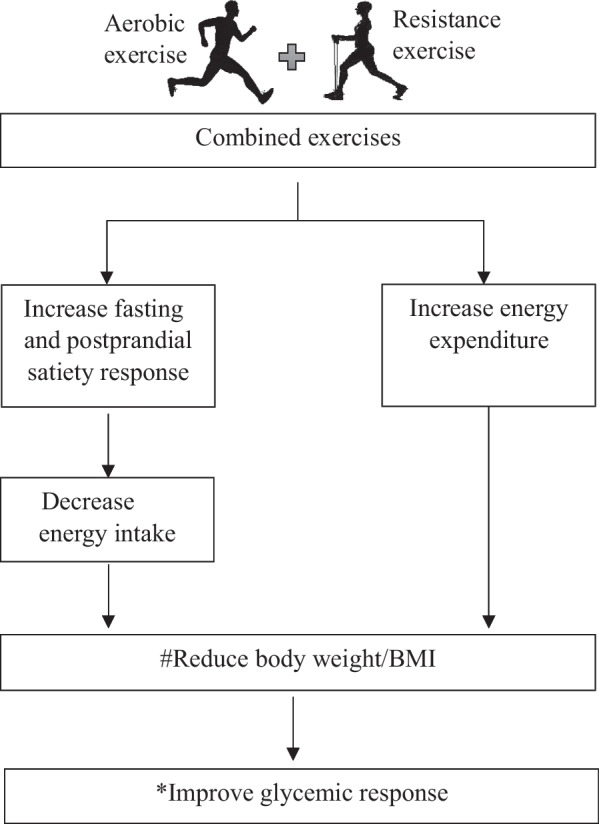
As indicated by Bonferroni post-hoc results, the interaction of time × intervention was significant between aerobic vs combined (*p* = 0.018) and combined vs control (*p* = 0.001) for perceived satiety at 60 min after a standard meal (aerobic n = 36, combined n = 34). Two-way repeated-measures ANOVA followed by Bonferroni post-hoc showed that the interaction of time × intervention for energy intake was significant between intervention and control groups, where both exercise groups showed reduced energy intake (aerobic vs control *p* = 0.021, combined vs control *p* = 0.015) compared to controls. The figure depicts that the reduction of #bodyweight/BMI [32] and the reduction of *HbA1c occur due to increased energy expenditure [33] and decreased energy intake following combined exercises. The contribution of combined exercises to perceived satiety and energy intake is indicated

Furthermore, the Bonferroni post-hoc results indicated that the interactions were significant between aerobic vs combined: − 9.4 mm, CI = − 1.2, 17.6, *p* = 0.018 and combined vs control: 12.6 mm, CI = 4.4, 20.8, *p* = 0.001 for satiety 60 min after the meal where the combined exercise group reported the highest ratings for satiety on VAS (41.76 mm) over other 2 groups at 6 months (Fig. 3).

As a previous study reported that the mean calorie intake of a Sri Lankan individual with T2DM is around 1438 kcal/day [31], we assumed that our study participants had a calory surplus diet at the baseline (Aerobic group—1736 kcal, Combined group—1781 kcal, Control group—1941 kcal). Although the mean calorie intake/day is comparatively higher among the participants of the present study, there was no significant difference observed between groups (*p* = 0.571).

As clearly evident in Table 4, the energy (median/IQR) and carbohydrate intake (median/IQR) at 3 and 6 months in people with T2DM who performed combined exercises were the lowest.

**Table 4:** Comparison of median differences of total energy and macronutrient intake between baseline, 3 and 6 months in aerobic and combined exercise groups with the controls

	Aerobic (AE) (n = 36)	Combined (COM) (n = 34)	Control (CON) (n = 36)	*p* value		
				AE vs CON	COM vs CON	AE vs COM
^#^*Differences (Δ) median/IQR between 0 and 3 months*						
Energy intake (kcal)	− 169.1 (47.3)	− 172.2 (35.7)	0.0 (9.0)	0.313	0.026*	0.949
Carbohydrate (g)	− 30.3 (8.3)	− 30.3 (8.0)	0.0 (5.8)	0.079	0.039*	0.768
Protein (g)	+ 3.1 (2.9)	+ 2.4 (2.8)	− 10.6 (4.9)	0.133	0.118	0.842
Fat (g)	− 0.4 (1.6)	+ 1.7 (7.7)	+ 7.3 (6.9)	0.001*	0.002*	0.337
^¥^*Differences (Δ)median/IQR between 0 and 6 months*						
Energy intake (kcal)	− 76.0 (2.5)	− 106.0 (39.3)	− 6.0 (4.3)	0.012*	0.022*	0.884
Carbohydrate (g)	− 50 (0.0)	− 50.0 (14.0)	− 13.5 (5.0)	0.001*	0.001*	0.548
Protein (g)	0.0 (20.7)	+ 1.1 (10.7)	0.0 (15.7)	0.197	0.193	0.957
Fat (g)	0.0 (5.5)	0.0 (6.3)	+ 9.5 (9.0)	0.001*	0.007*	0.674

Compared by Kruskal–Wallis test with post-hoc, *p < 0.05

^#^Values were obtained by deducting the baseline value from the 3 months

^¥^Values were obtained by deducting the baseline value from the 6 months

Two-way repeated-measures ANOVA showed that there was a significant time × group interaction (Wilk’s Lambda) [F (4, 204) = 5.6, *p* = 0.001] for the energy intake. However, none of the time × group interactions were significant for carbohydrate, protein and fat intakes.

Bonferroni post-hoc results showed that the interaction of time × intervention for energy intake was significant between intervention and control groups, where both exercise groups showed reduced energy intake (aerobic vs control: − 287.8 kcal, CI = − 34, 542, *p* = 0.021, combined vs control: − 304.3 kcal, CI = − 46, 562, *p* = 0.015) compared to controls with the lowest mean daily energy intake of 1571 kcal observed in the combined exercise group at 6 months compared to other 2 groups (aerobic—1711 kcal, control—1986 kcal). In the control group, no change was observed in energy intake at the end of 6 months, compared to their baseline (baseline vs 6 months, − 45 kcal, CI = − 15.8, 20.3, *p* = 0.243).

At 3 months, the body weight was significantly reduced in aerobic and combined exercise groups compared to controls (aerobic vs control, − 3.8 kg, CI = − 7.5, − 1.1, *p* = 0.027 and combined vs control, − 3.8 kg, CI = − 7.4, − 1.2, *p* = 0.038) and at 6 months a significant reduction in the body weight was observed in the aerobic and combined exercise groups compared to controls (aerobic vs control, − 4 kg, CI = − 7.6, − 1.3, *p* = 0.001 and combined vs control, − 3.8 kg, CI = − 7.4, − 1.3, *p* = 0.001).

At 3 months, the BMI was significantly reduced in aerobic and combined exercise groups compared to controls (aerobic vs control, − 2.4kgm^2^, CI = − 4.4, − 0.5, *p* = 0.005 and combined vs control, − 1.3 kg m^2^, CI = − 3.2, − 0.4, *p* = 0.009) and at 6 months also the aerobic exercise group and combined exercise group showed reduced BMI compared to controls (aerobic vs control, − 2.4 kg m^2^, CI = − 4.3, − 0.5, *p* = 0.003 and combined vs control, − 1.9 kg m^2^, CI = − 3.8, − 0.9, *p* = 0.002). At the outset, although participants in the intervention groups were overweight/obese whereas, toward the end of the study, the majority of participants in the intervention groups achieved normal weight (i.e., 63% in the aerobic group, 72% in the combined group).

Two-way repeated-measures ANOVA indicated that there was a significant time × group interaction (Wilk’s Lambda) [F (4,196) = 17.5, *p* = 0.001] on body weight.

Bonferroni post-hoc results showed that the effect of the intervention on body weight was significantly different between groups (aerobic vs control, − 3.9 kg, CI = − 7.6, − 0.3, *p* = 0.026 and combined vs control, − 3.6 kg, CI = − 7.3, − 0.1, *p* = 0.05), where both intervention groups showed reduced body weight with exercises compared to controls. The body weight in the control group was significantly increased at 6 months, compared to their baseline (baseline vs 6 months, − 3.6 kg, CI = − 5.1, − 2.0, *p* = 0.001).

Similarly, the findings of two-way repeated-measures ANOVA showed that there was a significant time × group interaction (Wilk’s Lambda) [F (4,198) = 3.8, *p* = 0.005] on BMI in the study participants.

Bonferroni post-hoc results showed that the effect of the intervention on BMI was significantly different between groups (aerobic vs control, − 2.3 kg m^2^, CI = − 4.2, − 0.4, *p* = 0.012 and combined vs control, − 1.8 kg m^2^, CI = − 3.8, 0.4, *p* = 0.05) where both intervention groups showed reduced BMI with exercises compared to controls. The BMI in the control group was significantly increased at 6 months, compared to their baseline (baseline vs 6 months, − 0.3 kg m^2^, CI = − 0.5, − 0.7, *p* = 0.01).

**Fig. 4:**
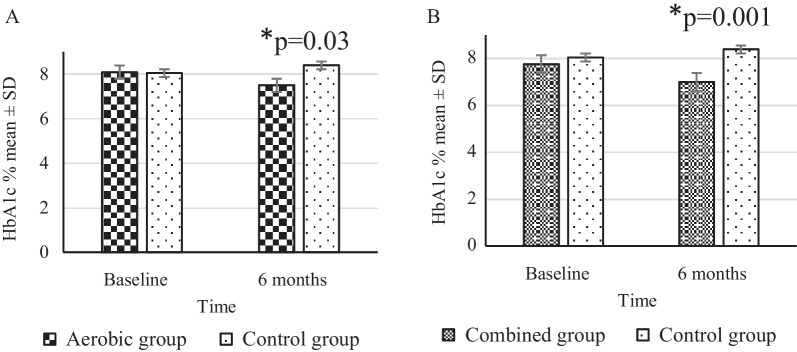
Absolute values of HbA1c % (mean ± SD) between groups at baseline and 6 months, compared by Tukey post-hoc test (aerobic n = 36, combined n = 34). **A** indicates reduced HbA1c% in the aerobic group compared to controls (*p* = 0.03), and **B** indicates reduced HbA1c% in the combined group compared to controls, at 6 months (*p* = 0.001), *p < 0.05

As shown in Fig. 4, there was a significant reduction in the HbA1c at the end of 6 months in both intervention groups compared to the control group implying an improvement in the glycemic response with aerobic and combined exercises (Fig. 4A, B). Further analysis in two-way repeated-measures ANOVA revealed that there was a significant time × group interaction (Wilk’s Lambda) [F (2,101) = 7.2, *p* = 0.001] for HbA1c levels.

Bonferroni post-hoc results showed that there was a significant impact of time × intervention between aerobic vs control: − 0.4%, CI = − 1.1, − 0.2, *p* = 0.025 and combined vs control: − 1.2%, CI = − 1.8, − 0.5, *p* = 0.001 where both exercise groups showed significantly reduced HbA1c levels compared to controls. No change in HbA1c was observed in the control group at 6 months, compared to their baseline (baseline vs 6 months, − 0.34%, CI = − 1.1, 0.4, *p* = 0.16).

## Discussion

To our understanding, the present study is one of the few studies that investigated the impact of regular exercises on appetite markers (hunger and satiety) and energy intake. The assessment of appetite markers following combined exercises (aerobic and resistance together) in people with T2DM is a novel aspect studied.

A previous study by Molfino et al. [34] has used 05 different tools to assess appetite in humans i.e., self-assessment of appetite, subjective assessment of appetite, visual analogue scale (VAS), Functional Assessment of Anorexia/Cachexia Therapy (FAACT) score and the Anorexia Questionnaire (AQ). Among the 05 tools, VAS has been identified as the best tool to assess the present appetite sensations in individuals. Furthermore, Molfino et al. [34] stated that the VAS is particularly useful in daily practice in clinical studies since it takes only a few seconds to perform, does not require specific competencies, and is associated with recent food intake.

The validity and the reliability of the visual analogue scale in the measurement of appetite have been accepted and, the scale has been used extensively in research studies [24]. Thus, in this study, the subjective level of hunger and satiety were quantified using the 100 mm visual analogue scale.

We hypothesized that long-term regular exercises alter appetite markers in people with T2DM. In this investigation, the appetite markers were assessed in relation to a standard breakfast meal while the energy intake by a 3-day diet dairy.

In the present study, a reduction in pre- and post-meal hunger levels was observed in both intervention groups compared to the control group at 3 and at 6 months. However, there was no significant difference in the hunger level between the two exercise groups at both time points. Further, a significant increase in pre- and post-meal satiety levels was observed in the combined exercise group compared to controls whereas only the pre-meal satiety was shown to increase in the aerobic exercise group following exercises for 3 and 6 months. Interestingly, the Bonferroni post-hoc results indicated a significantly higher time × group interaction for satiety (post-meal) in the combined exercise group compared to the aerobic group.

It is already known that the impact of exercises on appetite markers defers according to the type, volume, intensity, and duration of the exercise regimen [13]. Although changes in appetite markers in healthy normal weight and obese individuals following aerobic and resistance exercises have been studied extensively [13, 35], similar investigations on people with T2DM are limited.

Previous experiments on changes in appetite markers following acute exercises showed varying results. A study on healthy normal-weight males following single bouts of aerobic and resistance exercises has shown a reduction in the level of hunger with both types of exercises [36]. However, in another study in a similar group of participants, no changes in appetite markers were observed following a single bout of aerobic exercise compared to their pre-exercise control trials [37]. Although limited data are available on the assessment of appetite markers following combined exercise regimes, no change in hunger or satiety was noted in overweight healthy men undergoing single bouts of exercise regimens with combined aerobic and resistance exercises [35].

In previous studies of obese people with T2DM, single bouts of aerobic [12] and resistance [38] exercises were noted to decrease the perceived level of hunger and increase the perceived level of satiety implying that acute exercises irrespective of the type, have the ability to alter appetite markers in people with T2DM. Although the exercise-induced changes favor reduced food intake, these effects are reported to be transient [12]. The reduced hunger and increased satiety observed for a longer duration in the current study are possibly attributable to the regular and continuous nature of the exercises performed by the participants.

Although there are investigations observing the effect of long-term exercises on appetite markers in healthy adults, there is a dearth of information on studies conducted on people with T2DM. In most available reports in healthy adults, the exercise regimens seem to have one type of exercise carried out for up to a duration of 12 weeks [37, 39]. Although assessing gut hormones were beyond the scope of our study, the basis of the exercise-induced alterations in appetite markers may be explained by the changes in the gastrointestinal hormonal milieu [40].

A recent study by Vasto et al. [41] showed that 20 weeks of aerobic exercises increase gut hormones i.e., glucagon-like peptide-1 (GLP-1) and glucose-dependent insulinotropic polypeptide (GIP) in healthy individuals. Previous studies have reported the possible mechanisms of appetite regulation and showed that appetite control is modulated through hormones such as ghrelin, GLP-1 and gut hormone peptide YY (PYY) in healthy [36] as well as in people with T2DM [42]. These mechanisms cannot be discussed in detail since we have not performed hormonal assessments in our study participants. A previous study has shown that 12-week high-intensity aerobic exercises increased the level of satiety hormone GLP-1 compared to low-intensity aerobic exercises in patients with T2DM [43]. In addition, a recent investigation conducted on obese patients with T2DM who took part in 12 weeks of high-intensity aerobic exercises reported a reduction in ghrelin concentration and increased PYY and GLP-1 concentrations attributing to an increase in satiety [44].

Healthy food habits constitute a core element in the self-management of T2DM. Although studies investigating the relationship between exercises and food consumption in healthy individuals are immense [45, 46], according to our understanding there are no published reports on regular long-term exercises on food consumption in people with T2DM. Most previous studies have used a standard meal to assess food consumption following regular exercises [16, 37] whereas, in the present study, the results seem to be more authentic as the food consumption was based on the data of a 3-day diet diary which gives an accurate reflection of the normal meal pattern of the study participants [26].

According to our results, in our cohort of people with T2DM, a similar significant reduction of energy intake was observed in both exercise groups, irrespective of the exercise regimen they followed. However, a previous study of obese subjects with T2DM who performed single bouts of aerobic exercises failed to show any significant change in the post-exercise energy intake [12]. Moreover, overweight healthy women who performed aerobic exercises for 18 months showed no change in energy and macronutrient intake compared to their baseline [32]. The exact reasons for these findings are not yet understood. In addition to the changes in appetite markers, as suggested by our findings, alteration in the liking for specific food types induced by regular physical activity [10] is another likely factor that contributes to the changes in energy intake. We also presume that the behavior of satiety hormones contributes to the differences evident in energy intake following long-term exercises between healthy vs people with T2DM. Although 3 months of regular aerobic exercises were able to increase the PYY and GLP-1 levels in obese patients with T2DM [44], neither aerobic nor resistance exercises for the same duration were able to alter them in obese healthy men [39].

In addition, the combined exercise regimen was shown to have a faster effect on reducing energy intake compared to the aerobic regimen. It is interesting to note that the magnitude of the reduction in energy and macronutrient intake between baseline and 6 months in the aerobic group was achieved in the combined exercise group at 3 months. Thus, it is likely that combined exercises elicit a greater impact on energy and macronutrient intake within a shorter duration compared to the aerobic-only regimen. This is indeed another novel finding in our study which proves the greater efficacy of combined exercises in the reduction of energy intake compared to aerobic exercises. This observation can be further strengthened by the significantly higher level of satiety shown in the combined exercise group at 3 months compared to the aerobic group which has probably driven to reduce the energy intake.

It is well established that exercises reduce body weight and BMI due to energy expenditure [32]. In the current study, regular exercises for durations of 3 and 6 months were able to reduce the body weight and BMI in people with T2DM. Similar findings are reported in non-diabetic overweight/obese individuals who performed aerobic exercises for 12 weeks as well [47, 48]. In a study conducted on Japanese women, the decrease in body weight following 12 weeks of aerobic exercises was paralleled with increased secretion of the satiety hormone, GLP-1 [49], implying that exercises not only influence energy expenditure but also indirectly modulate energy intake as well. As supported by previous evidence, the reduction in the body weight and BMI in the present study may thought to be contributed by the anorexic effect of the exercise induced increase in the secretion of satiety hormones [47, 48]. Thus, we postulate that, besides energy expenditure, the reduction in energy intake also contributes in weight reduction in people with T2DM.

Glycemic control is one of the main goals of regular exercises in people with T2DM. In the present study, long-term regular exercises of either aerobic or combined type were able to significantly lower the HbA1c level when compared to baseline and also when compared to controls. Also, the HbA1c levels achieved by the subjects who performed combined exercises were comparatively lower than the ones who performed the aerobic type. Although the differences obtained between the two exercise groups in the present study did not show statistical significance, a recent systematic review highlights that combined exercises produce a pronounced improvement in the glycemic response when compared with either aerobic or resistance exercises alone [5]. On linking glycemic and appetite markers, a previous study has shown a significant positive correlation between perceived satiety and glycemic control in people with T2DM [50] possibly through satiety hormones such as GLP-1. However, the exact contribution of satiety on glycemic response is not certain since the increase in insulin sensitivity also plays a significant role in glycemic control following exercises [33]. Thus, the present study proposes increased satiety as another plausible explanation for the reduction in HbA1c in persons with T2DM who engage in regular aerobic and combined exercises.

The already proven fact is that energy expenditure in regular exercises contributes in reducing the BMI and HbA1c in people with T2DM. In the present study, no difference in the energy intake at baseline assessed by 3-day diet diary was noted in both exercise groups. As shown by the Bonferroni post hoc test, the subsequent reductions in the energy intake in the combined exercise group compared to the control group can be attributed to the impact of exercise. We believe that even in the aerobic group engaging exercise for a longer duration might show reductions in energy intake. This strongly implies that exercises influence energy intake as well. This was even proven in previous experiments carried out in healthy adults [13, 51]. Therefore, the reduced BMI and HbA1c in the intervention groups may be attributed not only to energy expenditure but also to the exercise-induced satiety response in people with T2DM.

In summary, regular exercises, either aerobic alone or combined with the resistance type are shown to facilitate glycemic control in many ways in persons with T2DM. However, the present study has proven extra advantages of a combined exercise regimen over an aerobic-only schedule due to its significant impact on increasing the level of satiety and the faster reduction of energy intake. Although the findings are shared with the aerobic group, the combined exercises also showed reduced hunger, BMI and HbA1c compared to inactive people with T2DM. Hence, the present study emphasizes the need for people with T2DM in engaging in regular exercises, preferably the combined type.

### Strengths and limitations

One important strength of this study is the ability to distinguish the changes in appetite markers observed between long-term aerobic and combined exercises. Most studies in this field were carried out with a limited number of healthy individuals over a period of 12 weeks, whereas in this study a cohort of people with T2DM who participated in regular exercises over a period of 6 months was assessed. Most studies of this nature were carried out testing resistance or aerobic-only exercise sessions, whereas we observed appetite markers, energy and macronutrient intake, body weight/BMI and glycemic response in people with T2DM who were performing combined exercises as well. Participants were not enforced to take a diet prescribed by the investigators and that helped in determining the influence of actual daily energy intake following exercises. In most studies, the absolute values were compared whereas in this study the net changes were calculated by deducting the baseline values with the data obtained at 3 and 6 months. Although the absolute values of HbA1c % were analyzed, the mean ± SD values were more or less equal to the glycemic status of the intervention and the control group at baseline. To standardize the appetite levels in the intervention and the control group, a standard meal was provided to both parties.

Restrictions on routine day-to-day activities in both intervention and control groups were not practical in this prospective study design. Routine physical activities [52] of mild to moderate intensity i.e., household activities, washing clothes, and walking, etc. carried out by the intervention and the control groups assessed at the baseline (at the time of recruitment), were found to be comparable. Thus, it was assumed that there was no significant difference in energy expenditure by routine physical activities between the two groups.

The hunger and satiety ratings were not taken at frequent time points to reflect long-term changes in appetite markers in the participants. It would have been ideal if we could obtain more ratings at frequent time points other than baseline, at 3 and at 6 months.

## Conclusions and recommendations

The present study is the first to examine the changes in hunger, satiety, energy and macronutrient intake in a larger cohort of people with T2DM who performed regular aerobic and combined exercises over a period of 6 months. Since both groups showed reduced hunger levels irrespective of the intervention, it is concluded that the type of exercise does not have a major impact on perceived hunger levels in people with T2DM.

The changes observed in satiety were more pronounced in those who performed combined exercises compared to aerobic exercise as well as compared to controls. Since the absolute values, as well as the differences between baseline with 3 and 6 months of the satiety levels of the combined exercise group, and the satiety levels of the combined were higher compared to aerobic and controls, we conclude that combined exercises can exert a greater impact on satiety levels in people with T2DM while having a faster effect on reducing the energy intake compared to the aerobic-only regimen.

Despite the complexity of the factors which affect appetite, it is imperative to understand the changes in appetite markers in people with T2DM who perform long-term exercises. Thus, future experiments should focus on changes in appetite markers and appetite-related hormones, particularly GLP-1 in long-term combined exercises and explore their contribution to increasing insulin sensitivity.

## Data Availability

The data used to support the findings of this study could be obtained from the corresponding author upon reasonable request.

## Abbreviations

ACSM: American College of Sports Medicine
ANOVA: Analysis of variance
AQ: Anorexia questionnaire
BMI: Body mass index
CI: Confidence interval
CONSORT: Consolidated Standards of Reporting Trials
ECG: Echocardiogram
FAACT: Functional Assessment of Anorexia/Cachexia Therapy
GLP-1: Glucagon-like peptide 1
GIP: Glucose-dependent insulinotropic polypeptide
HbA1c: Glycated haemoglobin
IQR: Interquartile range
PYY: Gut hormone peptide YY
SD: Standard deviation
SLCTR: Sri Lanka Clinical Trial Registry
T2DM: Type 2 diabetes mellitus
VAS: Visual analogue scale
